# Supplementary figures and images for: REDf: a deep learning model for short-term load forecasting to facilitate renewable integration and attaining the SDGs 7, 9, and 13
Source: PeerJ Comput Sci. 2025 Apr 23;11:e2819. doi: 10.7717/peerj-cs.2819 (PMC12190472; doi:10.7717/peerj-cs.2819)

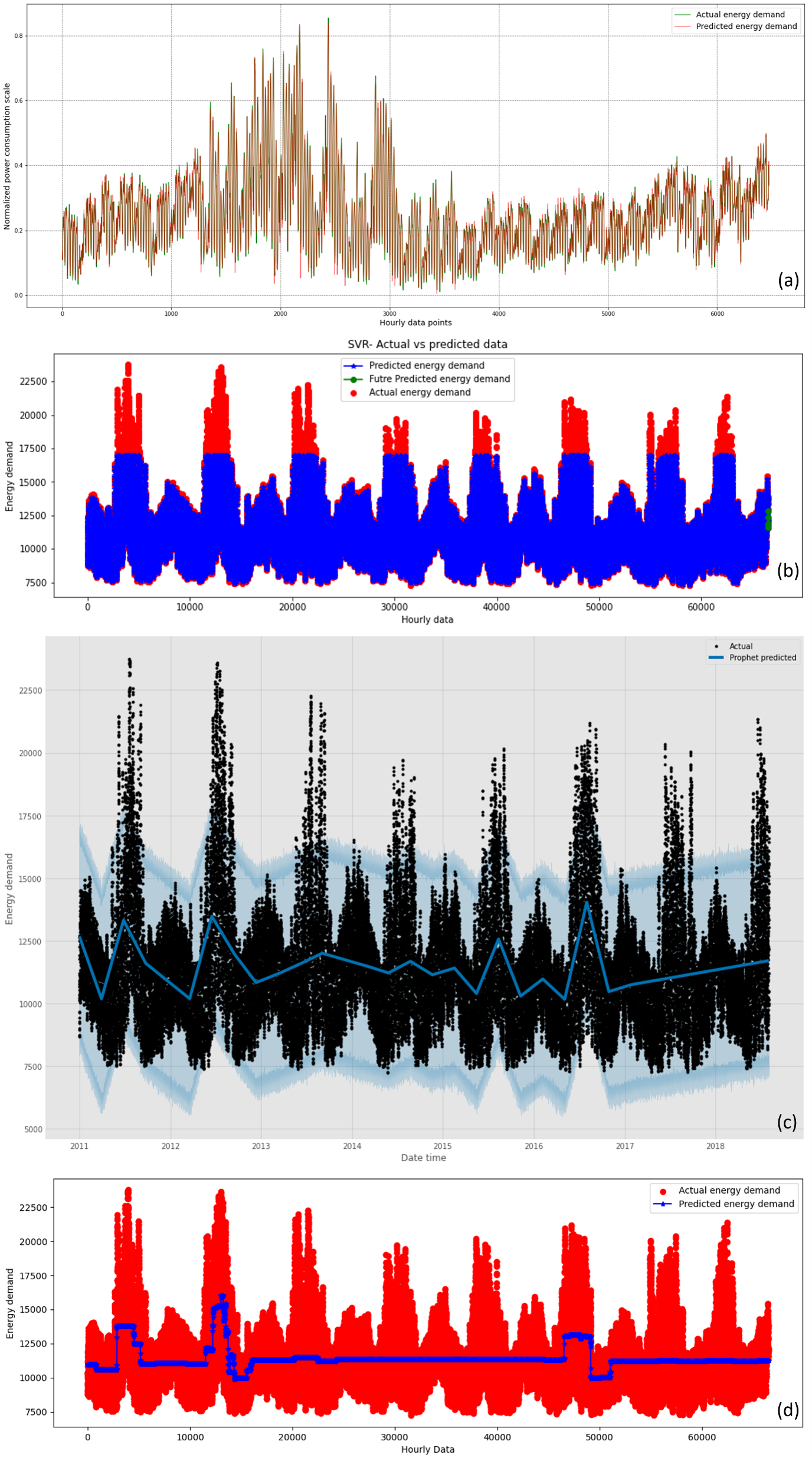

Supplement: Supplemental Information 1 — (A) Proposed REDf model, (B) SVR Model, (C) Facebook Prophet model, and (D) RFR model. [file peerj-cs-11-2819-s001.png]

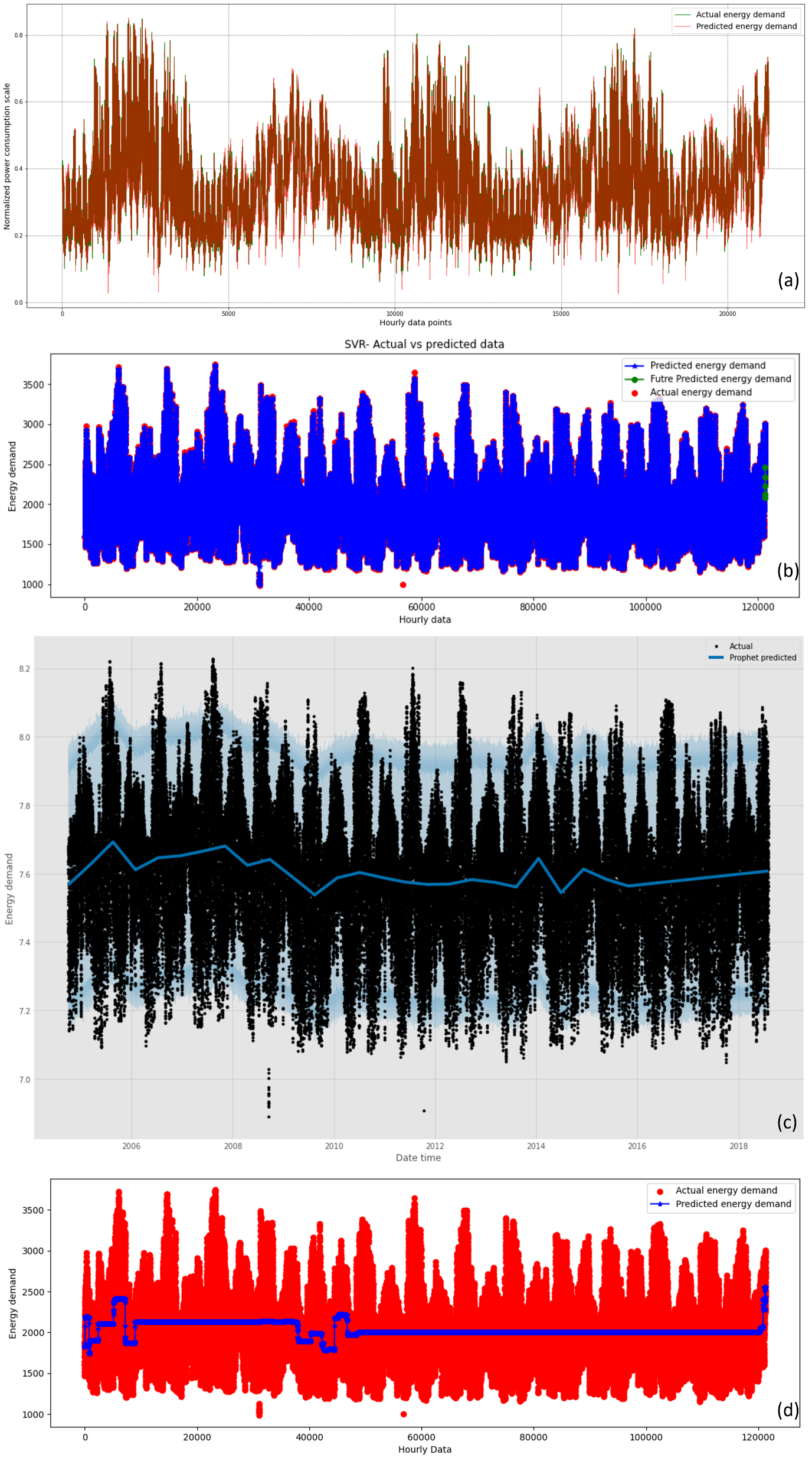

Supplement: Supplemental Information 2 — (A) Proposed REDf model, (B) SVR Model, (C) Facebook Prophet model, and (D) RFR model. [file peerj-cs-11-2819-s002.png]

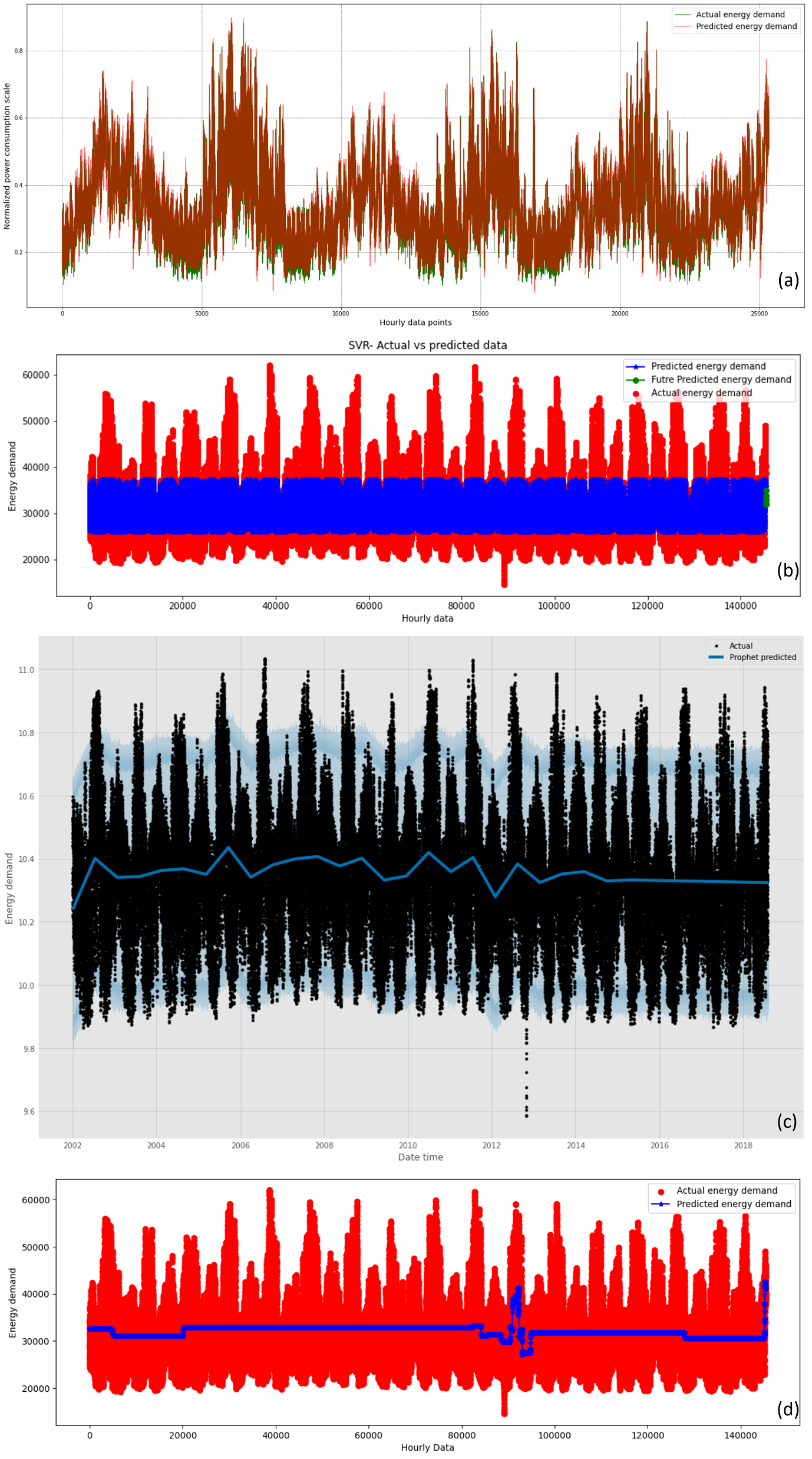

Supplement: Supplemental Information 3 — (A) Proposed REDf model, (B) SVR Model, (C) Facebook Prophet model, and (D) RFR model. [file peerj-cs-11-2819-s003.png]

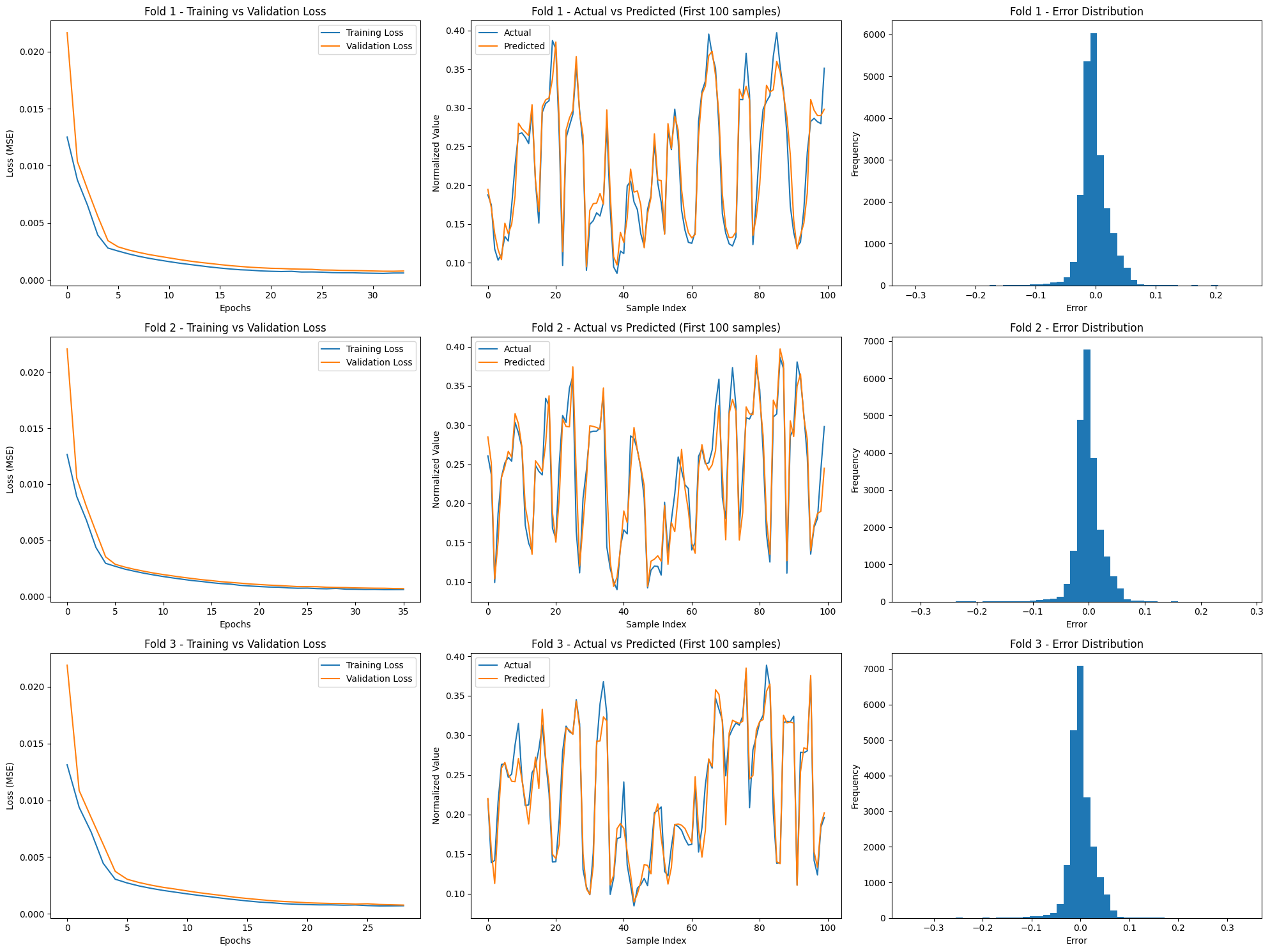

Supplement: Supplemental Information 4 — Each row corresponds to a different cross-validation fold. [file peerj-cs-11-2819-s004.png]

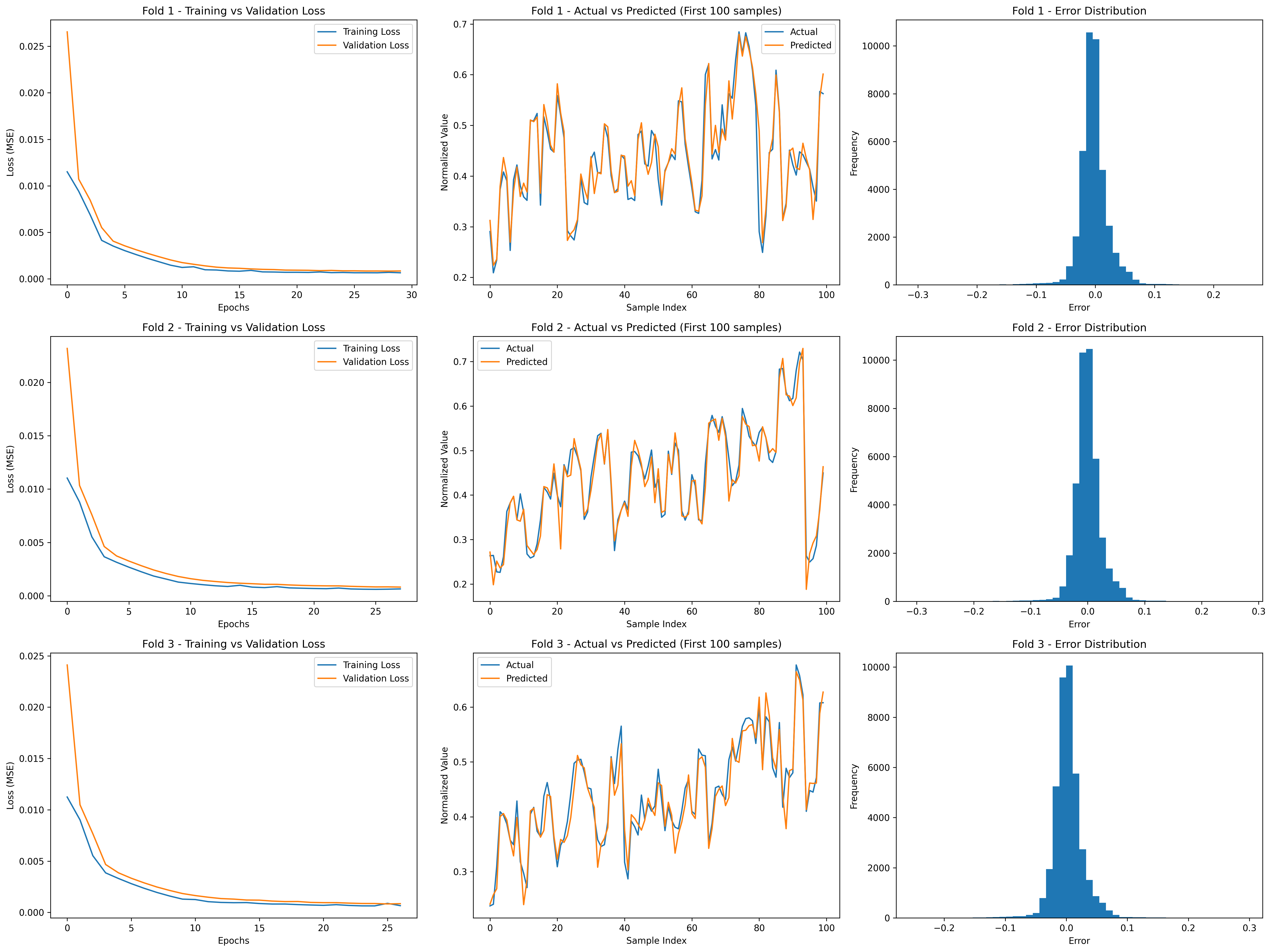

Supplement: Supplemental Information 5 — Each row corresponds to a different cross-validation fold. [file peerj-cs-11-2819-s005.png]

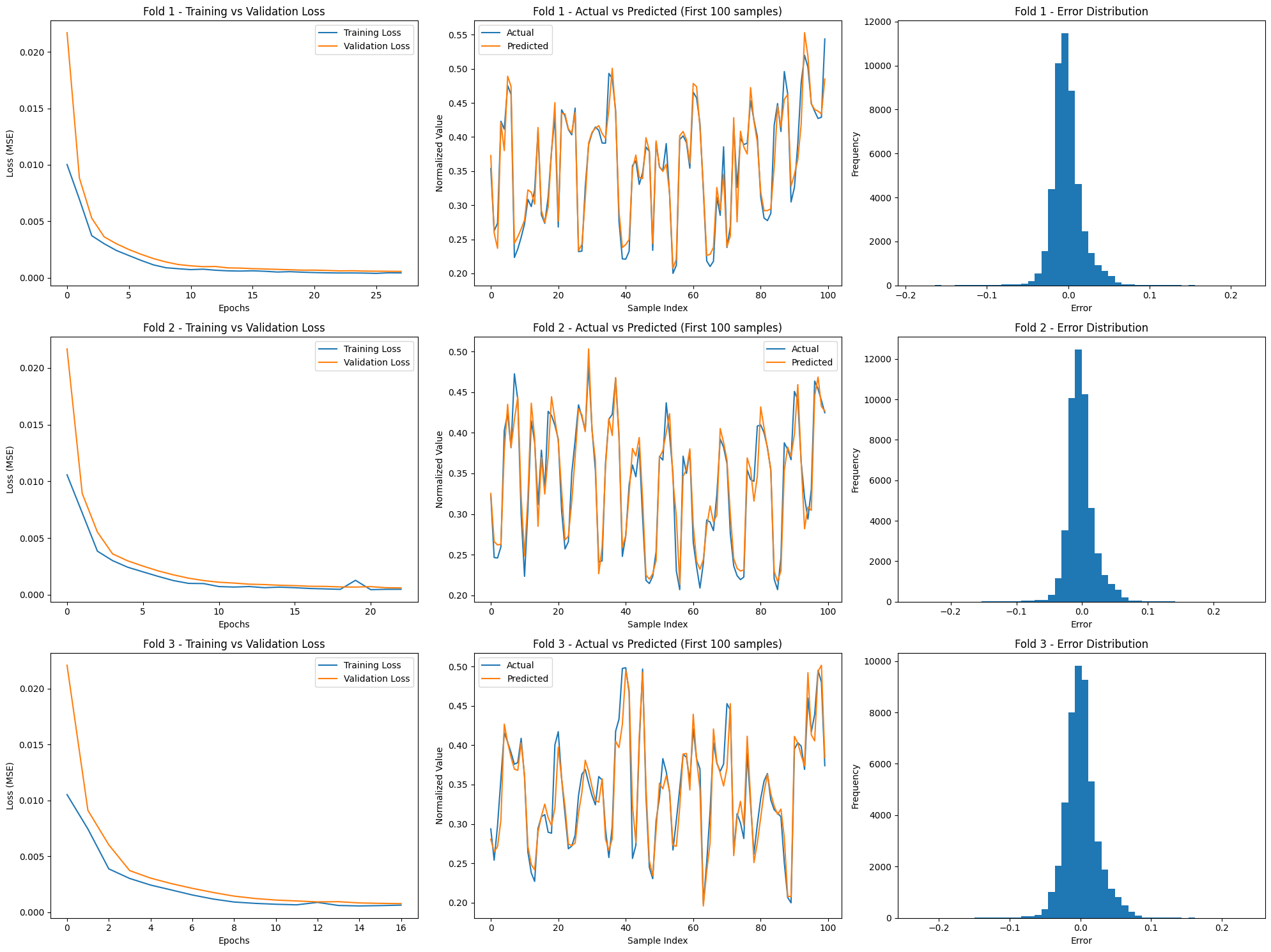

Supplement: Supplemental Information 6 — Each row corresponds to a different cross-validation fold. [file peerj-cs-11-2819-s006.png]
